# A Modified Kinematic Model of Shoulder Complex Based on Vicon Motion Capturing System: Generalized GH Joint with Floating Centre

**DOI:** 10.3390/s20133713

**Published:** 2020-07-02

**Authors:** Chunzhao Zhang, Mingjie Dong, Jianfeng Li, Qiang Cao

**Affiliations:** Beijing Key Laboratory of Advanced Manufacturing Technology, Faculty of Materials and Manufacturing, Beijing University of Technology, Beijing 100124, China; zhangchunzhao@emails.bjut.edu.cn (C.Z.); dongmj@bjut.edu.cn (M.D.); caoqiang@emails.bjut.edu.cn (Q.C.)

**Keywords:** glenohumeral joint, shoulder complex, Vicon motion capturing system, modified kinematic model, motion coupling

## Abstract

Due to the complex coupling motion of shoulder mechanism, only a small amount of quantitative information is available in the existing literature, although various kinematic models of the shoulder complex have been proposed. This study focused on the specific motion coupling relationship between glenohumeral (GH) joint center displacement variable quantity relative to the thorax coordinate system and humeral elevation angle to describe the shoulder complex. The mechanism model of shoulder complex was proposed with an algorithm designed. Subsequently, twelve healthy subjects performed right arm raising, lowering, as well as raising and lowering (RAL) movements in sixteen elevation planes, and the motion information of the markers attached to the thorax, scapula, and humerus was captured by using Vicon motion capturing system. Then, experimental data was processed and the generalized GH joint with floating center was quantized. Simultaneously, different coupling characteristics were detected during humerus raising as well as lowering movements. The motion coupling relationships in different phases were acquired, and a modified kinematic model was established, with the description of overall motion characteristics of shoulder complex validated by comparing the results with a prior kinematic model from literature, showing enough accuracy for the design of upper limb rehabilitation robots.

## 1. Introduction

Modeling shoulder motion is fundamental for understanding the dynamic behavior of upper limb, and the compatibility of the upper limb rehabilitation robot with the user is an urgent problem requiring resolution. If the kinematic model of the shoulder complex is not accurate, the designed upper limb rehabilitation robot will be incompatible with the upper limb of the users, and the connective interface of the exoskeleton will generate undesired interactional loads that are exceedingly detrimental to rehabilitation therapy. Therefore, accurate kinematic model of shoulder complex is very important to design the upper limb rehabilitation robot and it has practical significance to ensure the rehabilitation training effect of the affected limbs [1,2,3,4]. Yet, it is difficult to establish a complete kinematic model of the shoulder complex for it is a very complicated and correlative system [5]. In particular, the glenohumeral (GH) joint moves with the functional relevance of the shoulder girdle during humeral elevation [2]. Generally, the GH joint has three revolute degrees of freedom (DOFs) with the axes intersecting perpendicularly in the GH joint center, which is obviously a spherical joint [6]. The model of the shoulder girdle motion is far from simple, and its kinematic characteristics are crucial for remodeling the shoulder complex [5].

An effective way to solve the complexity of the shoulder girdle is through neglect and simplification [7]. Paper [8] presented a simple chain of shoulder movements, which only considered the GH joint as a centrally fixed 3-DOF spherical joint. Nevertheless, the GH joint and shoulder girdle are in a state of coupling motion during humeral elevation. The mean ratio of the GH joint to scapu-lothoracic motion is close to 1.7:1 [9,10,11]. Thus, neglecting the effect of the shoulder girdle has a serious impact on the movements of the shoulder complex. For the conjoint kinematics of the shoulder girdle, paper [12] presented a 5-DOF shoulder motion chain model that regarded the GH joint and the shoulder girdle as a 3-DOF spherical joint and a 2-DOF universal joint, respectively. Subsequently, to reflect the kinematic characteristics of the distance between the sternum and the GH joint center, paper [13] presented another 5-DOF shoulder motion chain model; in this model, the GH joint is equivalent to a 3-DOF spherical joint, and the shoulder girdle is equivalent to two prismatic joints whose axes are perpendicular to each other in the frontal plane. Paper [7] presented a 6-DOF shoulder motion chain model, in which the GH joint was considered as a 3-DOF spherical joint while the shoulder girdle was considered as a combination of a 2-DOF universal joint and a 1-DOF prismatic joint, respectively. To improve the kinematics and dynamics performance, [14] proposed a 7-DOF shoulder motion chain model; according to that model, the GH joint is equivalent to a 3-DOF spherical joint while the shoulder girdle is equivalent to a combination of a 3-DOF spherical joint and a 1-DOF prismatic joint. Paper [15] presented an 8-DOF shoulder motion chain model; in that model, the GH joint is equivalent to a 3-DOF spherical joint while the shoulder girdle is equivalent to a combination of a 3-DOF spherical joint and a 2-DOF universal joint. Paper [16] regarded the sternoclavicular (SC), the acromioclavicular (AC), and the GH joints as 3-DOF spherical joints with fixed centers, and these three joints were successively connected by two linkages to form a 9-DOF shoulder motion chain. Moreover, some researchers, who established the Maurel Model [17], Tondu Model [16], Berthonnaud Model [18], Lenarcic Model [19], as well as the Dynamic Model [20,21], considered the shoulder girdle in a closed-loop mechanism and tried to exactly copy the shoulder complex. Although establishing the shoulder kinematic model is the basis of quantitatively describing the movements of shoulder girdle during humeral elevation, it is not enough just to think about the mechanism model. The joint coupling motion relationship and the limiting of the coupling motion should also be considered [22,23,24].

Paper [23] studied the exact coupling motion relationship of the shoulder complex using the pin inserted into the sternum, clavicle, scapula and humerus, grouping the results as “gold standard” by collecting data to analyze the coupling relationship. Some other researchers studied the specific exact motion coupling relationship using the X-ray and computed tomography (CT) [24,25]. Unfortunately, the motion coupling relationship of the shoulder complex is in a certain particular position or defined plane. Moreover, there is no need to acquire accurate detection for each of the joint coupled motion of human upper limb [7,22]. Using motion capture technology to acquire detection data based on skin markers can meet the precision requirements of human daily movement science. Applying this technique, [22] obtained the position information of the GH joint center relative to different elevation angles and elevation planes by estimating the instantaneous rotation center of the GH joint through the average position of three marker points at each time. Based on a 6-DOF shoulder motion chain model of the shoulder complex, and the estimated motion information of the shoulder girdle and the GH joint center through the average position of two markers at each time, [7] synthesized the coupled motion between the movements of the shoulder girdle and the humeral elevation during humerus raising movement in four elevation planes (0°, 45°, 90°, and 135°). Since the complexity of the shoulder complex lies in the movement of shoulder girdle and the invisibility of the GH joint center [7,10,23], it is also difficult to quantify and understand the whole coupled motion of the shoulder complex [7,22]. Because humerus connects to the scapula of shoulder girdle through the GH joint [24,26], obtaining the GH joint center displacement variable quantity is equal to the overall motion of the shoulder girdle, and establishing the coupling motion relationship between the GH joint center displacement variable quantity relative to the thorax coordinate system and the humeral elevation can clearly describe the whole coupled motion of the shoulder complex.

In order to achieve this, further studies on the anatomical structure of the shoulder girdle and GH joint are necessary. The main contribution of the work is establishing a modified kinematic model of the shoulder complex based on Vicon motion capturing system, which can describe the specific motion coupling relationship between GH joint center displacement variable quantity relative to the thorax coordinate system and humeral elevation angle. The remainder of the paper is organized as follows. The mechanism model of the shoulder complex, an algorithm to depict the motion coupling relationship between the GH joint center displacement variable quantity relative to the thorax coordinate system and the humeral elevation angle, the motion-capture method, and postprocessing of experimental data are presented in Section 2. In Section 3, the motion coupling relationships in the raising, lowering, as well as raising and lowering (RAL) phases are described. The comparison of collected data with the Klopčar kinematic model and the differences in different phases of humeral elevation are discussed in Section 4. Finally, conclusion and future work are stated in Section 5.

## 2. Materials and Methods

### 2.1. Mechanism Model of the Shoulder Complex

**Figure 1:**
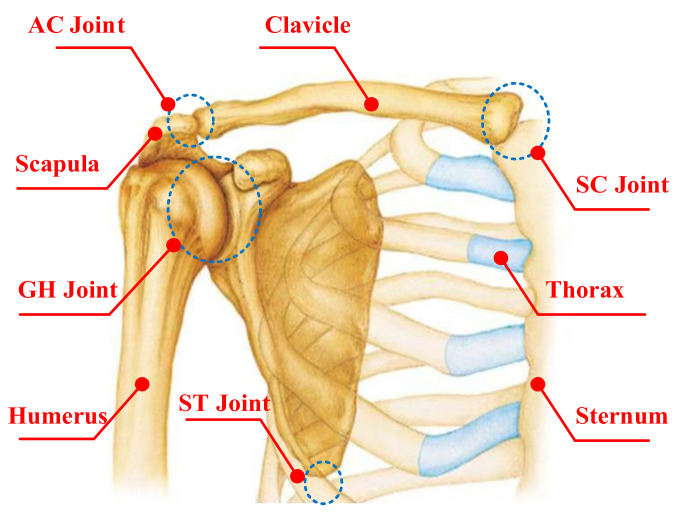
Anatomy of the shoulder complex.

Anthropotomy needs to be understood to model the shoulder’s complex [13,16]. The shoulder complex consists of the shoulder girdle and the humerus, and it includes four bones (the humerus, scapula, sternum, and clavicle) and four joints (the GH, SC, scapulothoracic (ST), and AC joints) [18,26], as shown in Figure 1 [27], which together perform the required functional motions and achieve maximum flexibility in the upper limb movement [28]. In general, the GH joint consists of the humeral head and the glenoid cavity of the scapula, which is usually equivalent to a spherical joint [26,29], and the kinematics of the AC, ST, and SC joints are not clear [30,31,32,33]. Thus, it is difficult to understand the motion characteristics of the shoulder girdle. Theoretically, all the joints and bones of the shoulder complex are particularly complex [25,34]. The mechanistic theory of joint physiology is helpful to understand the shoulder complex kinematics [16].

**Figure 2:**
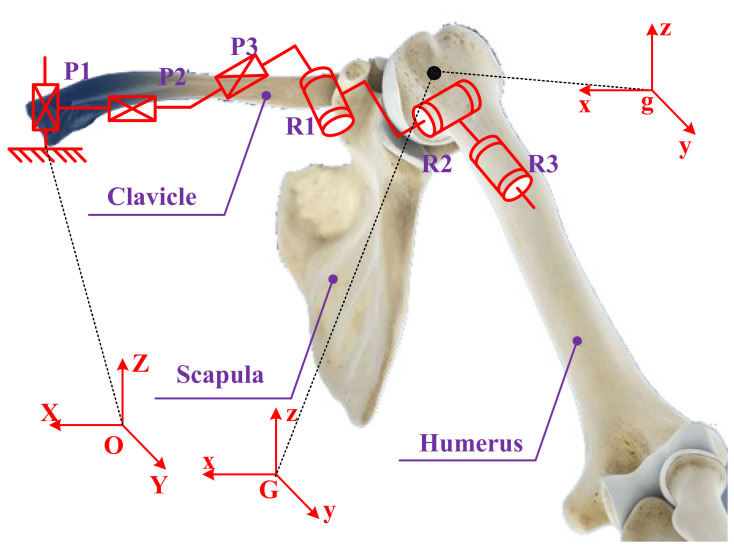
Mechanism model of the shoulder complex.

The humerus is connected to the scapula of shoulder girdle through the GH joint [26]. The GH joint has three revolute DOFs with the axes intersecting perpendicularly in the GH joint center and moves with the functional relevance of the shoulder girdle during humeral elevation, which is obviously a spherical joint [5,35]. Thus, the humerus has only three rotational motions around the GH joint center relative to the shoulder girdle. To expand on this point, the GH joint center can be considered as a virtual point on the shoulder girdle and a real point on the humerus head, and the two points coincide. According to the knowledge of theoretical mechanics, the general motion of a rigid body can be decomposed into translation following any of the base points and rotation relative to the base point. In exactly the same manner, the shoulder girdle is assumed to be a platform, and the GH joint center is considered to be the base point of the moving platform. The mobile reference frame G-xyz is established on the shoulder girdle, and the origin of the mobile reference frame *G* is a virtual point on the shoulder girdle coinciding with the GH joint center. Relative to the global coordinate system O-XYZ, the G point has three translational DOFs. The fixed connection coordinate frame g-xyz is established on the humerus head, and the origin g is a real point on the humerus head coinciding with the GH joint center. Relative to the mobile reference frame, the three rotational axes of humerus intersect perpendicularly in the g point. In this way, the motion of humerus can be equivalent to the general motion of a rigid body, except that the motion of the rigid body is subject to certain constraints. Thus, a mechanism model of the shoulder complex: a generalized GH joint with floating center (i.e., a 3-DOF spherical joint with floating center, whose center displacement variable quantity relative to the thorax coordinate system is coupled with its rotation) has been presented, as shown in Figure 2 [27].

### 2.2. Design of the Algorithm

The GH joint is equivalent to a spherical joint that connects the humerus and the scapula. Based on the joint center regression analysis, a method is characterized to measure the motion coupling relationship between the GH center displacement variable quantity relative to the thorax coordinate system and the humeral elevation angle by the following three steps.

Step (1): Acquiring the motion data of the shoulder complex. The skin-mounted markers are used to measure the shoulder complex by the detection system, and the method of pasting markers and naming rules are described in the “marker placements”. Through the establishment of the thorax coordinate system and coordinate transformation, the markers’ motion information of the scapula, humerus, lateral epicondyle (EL), and medial epicondyle (EM) can be expressed in the thorax coordinate system at any time during the measurement.

**Figure 3:**
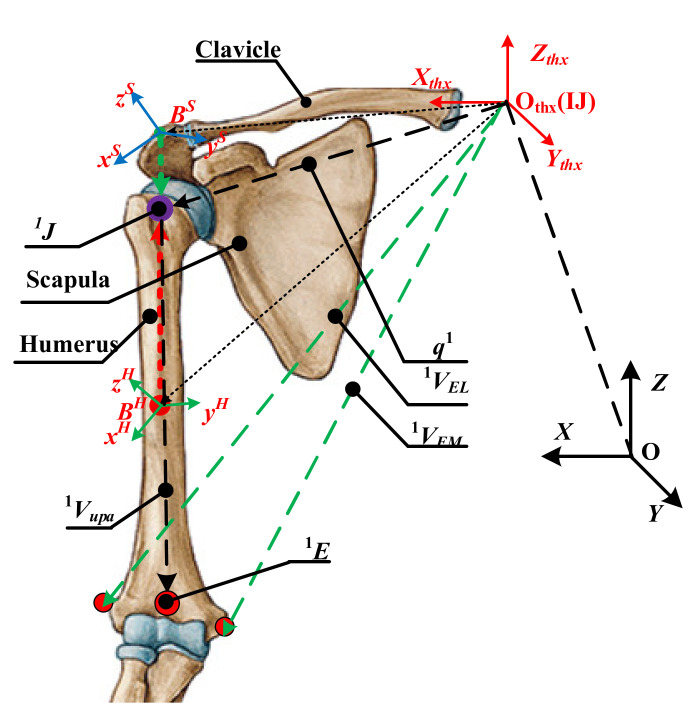
The model of the glenohumeral (GH) joint center and the humeral elevation angle.

**Figure 4:**
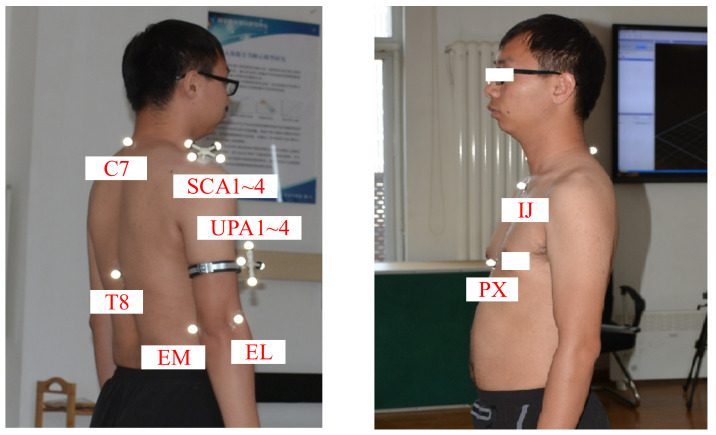
Marker placements of subject.

Step (2): Obtaining the GH joint center displacement variable quantity. The scapula and humerus are used as rigid bodies. Based on the theory of rotation geometry, a kinematic model with an invariant center related to the adjacent rigid body is established. Expanding on this point, the GH joint center is invariable in the coordinate frame fixed on the scapula and humerus. In addition, the respective frame is established by four markers of the block, as shown in Figure 3 and Figure 4. Based on the invariance of the GH joint center, the regression parameters are obtained and the GH joint displacement variable quantity can be calculated.

Step (3): Establishment of the motion coupling relationship. According to the method described by the International Society of Biomechanics (ISB) [36,37], the humeral elevation angle can be calculated. Thus, the motion coupling relationship between the GH joint center displacement variable quantity relative to the thorax coordinate system and humeral elevation angle can be obtained.

#### 2.2.1. Acquiring the Motion Data of the Shoulder Complex

According to the method and naming rules in ISB [36] ***C***7, ***T***8, ***IJ***, and ***PX*** are the position vector relative to the global coordinate frame, of which, ***C***7 represents the processus spinosus of the 7th cervical vertebra, ***T***8 represents the processus spinosus of the 8th thoracic vertebra, ***IJ*** represents the deepest point of incisura jugularis, ***PX*** represents the processus xiphoideus, the most caudal point on the sternum, and *IJ*_x_, *IJ*_y_, and *IJ*_z_ are the coordinate component of the ***IJ***. Thus, the thorax coordinate system is established with detection data markers according to (1).

(1) $$\left\{\begin{array}{l} x_{t}=(IJ-PX)\times (C7-PX)./\left|\left(IJ-PX)\times (C7-PX\right)\right|\\ z_{t}=(0.5*(IJ+C7)-0.5*(PX+T8))./\left|\left(0.5*(IJ+C7)-0.5*(PX+T8)\right)\right|\\ y_{t}=z_{t}\times x_{t},\;O_{t}=(IJ_{x},IJ_{y},IJ_{z}) \end{array}\right.$$

where *x_t_* direction is the cross product of vector ***IJ*-*PX*** and ***C7*-*PX***, and the length of *x_t_* is a unit vector which is obtained by unitization. *z_t_* direction is the midpoint vector of ***IJ*** and ***C7*** minus the midpoint vector of ***PX*** and ***T8***, and the length of *z_t_* is also a unit vector which is obtained by unitization. *y_t_* is the cross product of vector *z_t_* and *x_t_*. The specific principles can be referred to in [36]. All markers used to measure data are relative to the global coordinate frame, as in (2).

(2) x=(1,0,0), y=(0,1,0), z=(0,0,1), O=(0,0,0)

The matrix between the thorax coordinate system and global coordinate frame is defined according to (3).

(3) $$T^{O\to O_{t}}=\left[\begin{array}{cc} R_{3\times 3},\; & P_{1\times 3}\\ \;0, & \;1 \end{array}\right]=\left[\begin{array}{llll} x.x_{t}, & y.x_{t}, & z.x_{t}, & 0-IJ_{x}\\ x.y_{t}, & y.y_{t}, & z.y_{t}, & 0-IJ_{y}\\ x.z_{t}, & y.z_{t}, & z.z_{t}, & 0-IJ_{z}\\ \;0, & \;0, & \;0\;, & 1 \end{array}\right]$$

where *R*_3×3_ and *P*_1×3_ denote the rotation matrix and displacement vector, respectively.

Considering the wide motion ranges of the scapula and humerus, the markers may be occluded, leading to the fact that the location information of all the markers may not be captured completely. In general, the position and posture of a rigid body can be completely replaced by three markers that are not collinear. Therefore, three noncollinear markers’ data acquired from the four markers of the block fixed on the scapula and humerus are selected, respectively. Thus, the motion information of markers *P_i_*, which are *P*_1_, *P*_2_, and *P*_3_ of scapula, *P*_4_, *P*_5_, and *P*_6_ of humerus, *P*_7_ of EL, and *P*_8_ of EM, can be transformed into the thorax coordinate system at any time during the measurement according to (4), which are expressed as *P_ii_*.

(4) $$\left[\begin{array}{c} P_{ii}^{}\\ 1 \end{array}\right]=T^{O\to O_{t}}\left[\begin{array}{c} P_{i}^{}\\ 1 \end{array}\right]$$

#### 2.2.2. Obtaining the GH Joint Center Displacement Variable Quantity

As shown in Figure 4, *^K^P*_11_, *^K^P*_22_, and *^K^P*_33_ are the markers on the scapula, while *^K^P*_44_, *^K^P*_55_, and *^K^P*_66_ are the markers on the humerus, both of which are expressed relative to the thorax coordinate system. K represents the time during the measurement.

For the scapula, the regression parameters are *a*, *b*, and *c*, and the GH joint center *^K^J* relative to the thorax coordinate system can be derived from (5).

(5) $${}^{K}J=\left[{\;}^{K}q_{1}{\;}^{K}q_{2}{\;}^{K}q_{3}{\;}^{K}q_{4}\right]{\left[a\;b\;c\;1\right]}^{T}$$

where ${}^{K}q_{1}={\;}^{K}P_{11}{-}^{K}P_{22},{\;}^{K}q_{2}={\;}^{K}P_{11}{-}^{K}P_{33},{\;}^{K}q_{3}={(}^{K}P_{11}{-}^{K}P_{22}{,}^{K}P_{11}{-}^{K}P_{33}),{\;}^{K}q_{4}={\;}^{K}P_{11}$.

For the humerus, the regression parameters are *d*, *e*, and *f*, and the GH joint center *^K^J* relative to the thorax coordinate system can be derived from (6).

(6) $${}^{K}J=\left[{\;}^{K}q_{5}{\;}^{K}q_{6}{\;}^{K}q_{7}{\;}^{K}q_{8}\right]{\left[d\;e\;f\;1\right]}^{T}$$

where, ${}^{K}q_{5}={\;}^{K}P_{44}{-}^{K}P_{55},{\;}^{K}q_{6}={\;}^{K}P_{44}{-}^{K}P_{66},{\;}^{K}q_{7}={(}^{K}P_{44}{-}^{K}P_{55}{,}^{K}P_{44}{-}^{K}P_{66}),{\;}^{K}q_{8}={\;}^{K}P_{44}$.

Equation (7) can be obtained from (5) and (6), as follows.

(7) $$\left[{}^{K}q_{1}{\;}^{K}q_{2}{\;}^{K}q_{3}{\;}^{K}q_{5}{\;}^{K}q_{6}{\;}^{K}q_{7}\right].{\left[a\;b\;c\;d\;e\;f\right]}^{T}=-{(}^{K}q_{4}{-}^{K}q_{8})$$

Since multiple frames of data are collected continuously, the matrix equation can be obtained.

(8) AX=B

where,

$$A=\left[\begin{array}{ccccccc} {}^{1}q_{1}, & {\;}^{1}q_{2}, & {\;}^{1}q_{3}, & {\;}^{1}q_{5}, & {\;}^{1}q_{6}, & {\;}^{1}q_{7}\\ {}^{2}q_{1}, & {\;}^{2}q_{2}, & {\;}^{2}q_{3}, & {}^{2}q_{5}, & {\;}^{2}q_{6}, & {\;}^{2}q_{7}\\ \vdots & \vdots & \vdots & \vdots & \vdots & \vdots & \\ {}^{K}q_{1}, & {}^{K}q_{2}, & {}^{K}q_{3}, & {}^{K}q_{5}, & {}^{K}q_{6}, & {}^{K}q_{7} \end{array}\right],$$

$$X={\left[a,b,c,d,e,f\right]}^{T},\;B=\left[-{(}^{1}q_{4}{-}^{1}q_{8});-{(}^{2}q_{4}{-}^{2}q_{8});\cdots \cdots ;-{(}^{k}q_{4}{-}^{k}q_{8})\right]$$

The regression parameters are written as vector ***X***, which is obtained by solving (9).

(9) $$X={\left(A^{T}A\right)}^{-1}A^{T}B$$

After obtaining the vector ***X***, the GH joint center *^K^J* relative to the thorax coordinate system can be acquired by substituting it into (5) or (6). Then, the GH joint center displacement variable quantity *^KK^J* can be obtained by the GH joint center *^K^J* minus the *^1^J*.

#### 2.2.3. Establishment of the Motion Coupling Relationship

According to the method in ISB, the humeral elevation angle can be calculated through the humerus posture vector and ***Z***_thx_ axis of the thorax coordinate system, as shown in Figure 3.

Humerus posture vector:

(10) $${}^{K}V_{upa}=\frac{1}{2}{(}^{K}V_{EL}{-}^{K}V_{EM})-q^{K}$$

Humeral elevation angle:

(11) $$\theta^{K}=arccos(\frac{{}^{K}V_{upa}.z_{thx}}{\left|{}^{K}V_{upa}\right|.\left|z_{thx}\right|})$$

where *^K^**V**_EL_*, *^K^**V**_EM_,* and ***q****^K^*, which are *P*_77_, *P*_88_, and *^K^J* relative to the thorax coordinate system at *K* time, denote the posture vector of the EL, EM, and GH joint center respectively.

Based on the GH joint center displacement variable quantity *^KK^J* and the humeral elevation angle *θ^K^*, the motion coupling relationship between the GH joint center displacement variable quantity relative to the thorax coordinate system and the humeral elevation can be established.

### 2.3. Experiment Setup

Twelve participants were recruited from the staff and student populations of Beijing University of Technology. All participants (12 males; mean age 26 ± 6 years; arm length 551.5 ± 6.5 mm; height 174 ± 9 cm; weight 72 ± 12 kg) were healthy having no neurological or cardiopulmonary diseases. All experiments were approved by the Ethical Committee of Beijing University of Technology and conformed to the Declaration of Helsinki. The upper limb parameters of healthy subjects were measured and recorded in real time in the biomechanical laboratory.

#### 2.3.1. Marker Placements

Capturing the motion information of markers pasted on the skin is a common method to understand human movement. Considering the effect of skin deformation, the accuracy of measuring scapula and humerus is affected to some extent [7,22]. For humerus: to minimize the effect of skin deformation, the markers, which were placed on the upper arm by pressing the flange, were fixed to the flange. For scapula: [38] reported the slippage of the markers pasted on the acromion process was 4.2 mm during full elevation of the arm. Some researchers used the cluster of markers pasted on the acromion to reduce the effect of skin deformation [25,39]. Paper [40] limited the elevation angle within 150° to ensure the movement accuracy of the markers placed on the acromion when processing the motion data of the shoulder complex. Thus, in this experiment, the elevation angle is limited to 150°. The above method was used to paste the markers on the humerus and scapula.

Six skin-mounted markers were attached to the sternum, EL, and EM, as recommended by the ISB [36,37,39,40]. Specifically, four skin-mounted markers (***C7***, ***T8***, ***IJ*** and ***PX***) were used to calculate the thorax coordinate system. The origin of the thorax coordinate system was the ***IJ***. Two skin-mounted markers were located on the EL and EM, which were used to obtain the humeral posture vector. For the humerus, four markers of the block (UPA1-4), which were placed on the right upper arm by pressing the flange, were fixed to the flange. For the scapula, the other four markers of the block (SCA1-4) were placed on the acromion. The movement of the block is equivalent to the movement of the corresponding bone. To simplify the motion analysis, the description of the humerus and scapula do not follow the ISB completely [41,42], as shown in Figure 4.

#### 2.3.2. Experiment Protocol

**Figure 5:**
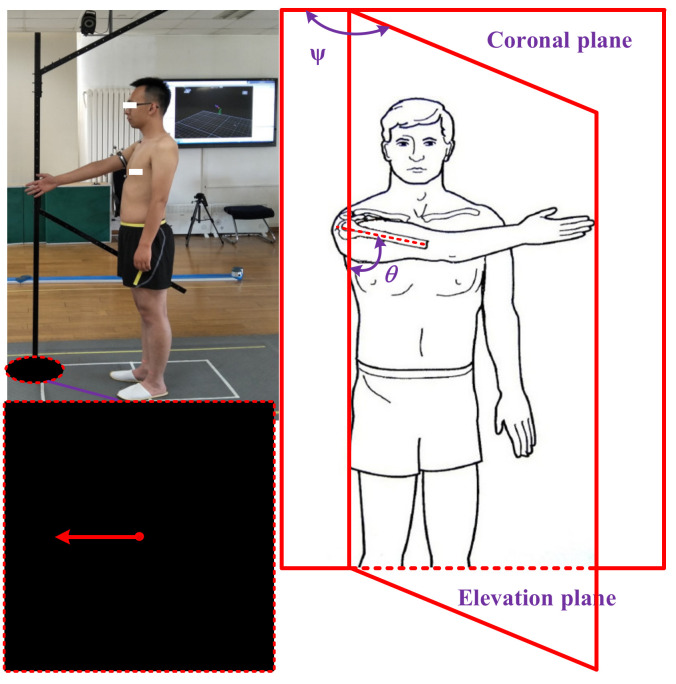
Elevation of the arm using the frame for guidance; guiding plane angle at 90°; description of experimental model at elevation plane angle η and elevation angle θ.

The experiment of collecting shoulder complex’s motion information was completed in the mechanics and biomechanics laboratory of the National Research Center for Rehabilitation Technical Aids, Ministry of Civil Affairs of the People’s Republic of China. The optical motion capture system VICON (Vicon Motion Systems Ltd., Oxford, UK) was adopted, which can detect the spatial information (3D data relative to the coordinate frame of VICON) of the markers placed on the participants. During the experiment, the sampling rate and the image resolution of the VICON system were adjusted to be 50 Hz and 1280 × 1024, respectively. The layout of the test site is shown in Figure 5. The gray part is the effective range that can be captured by the cameras, and the space height of the test site is 3.5 m.

The kinematic measurements of the shoulder complex were recorded with the subjects in the standing position, keeping their arms straight, relaxing the right shoulder hanging naturally, with no elbow flexion. Each subject performed raising, lowering, and RAL of the right arm, using the frame for guidance in the elevation planes, which included sixteen in total (0°, 10°, 20°, 30°, 40°, 45°, 50°, 60°, 70°, 80°, 90°, 100°, 110°, 120°, 130°, and 135°), and the elevation angle in different phases was from 0° to 150° (in order to obtain the elevation angle from 0° to 150°, the range of experimental elevation angle needs to be larger than 0° to 150°). All the subjects performed the motion three times.

### 2.4. Postprocessing of Experimental Data

After the experiment, the data obtained was brought into the formula which were presented in the “design of the algorithm”. We can obtain the motion coupling relationship in the raising, lowering, and RAL phases, respectively. Considering the different body structures of different subjects, normalization is a common method, which needs to determine the distance from the position of the ***IJ*** to the GH joint center when the subject’s arm was hanging naturally, and all the calculated data of the GH joint center were divided by the distance. The consistent approach was used in [7,22]. To sum up the relationship of motion law, it is necessary to obtain the motion mean curve of all individuals on all planes and to fit the mean value. However, there may be overfitting and underfitting phenomena during fitting [43,44]. The evaluation indexes are used to select the parameters of the fitting curve. In these indexes, the closer the sum of squares due to error (SSE) and root mean squared error (RMSE) are to zero, the better the model selection, fitting, as well as the data prediction will be. The closer the coefficient of determination (R-Square) and DOF adjusted coefficient of determination (Adjusted R-Square) are to one, the better the model fits the data. The general criteria (i.e., the R-Square and Adjusted R-Square are greater than 0.995; the SSE and RMSE are less than 0.005) are selected in the treatment of the average data, which is a similar method as used in [22].

## 3. Results

The experimental results indicated the motion coupling relationship between the GH joint center displacement variable quantity relative to the thorax coordinate system and the humeral elevation in the raising, lowering, and RAL phases, and the results were repeated with the same motion trend under the same conditions.

### 3.1. Motion Coupling Relationship in Different Phases

The elevation angles in the raising phase and lowering phase of humerus elevation are from 0° to 150° and from 150° to 0°, respectively, while the elevation angle in the RAL phase is from 0° to 150° and then from 150° to 0°. In order to show the regularity and conciseness of the results at different stages, the start to the end elevation angles in the experimental results are all set to vary from 0° to 150° (the experimental results have been processed to obtain the required elevation angle changing from 0° to 150°, so it does not show a peak-shape graph in lowering and RAL phases).

**Figure 6:**
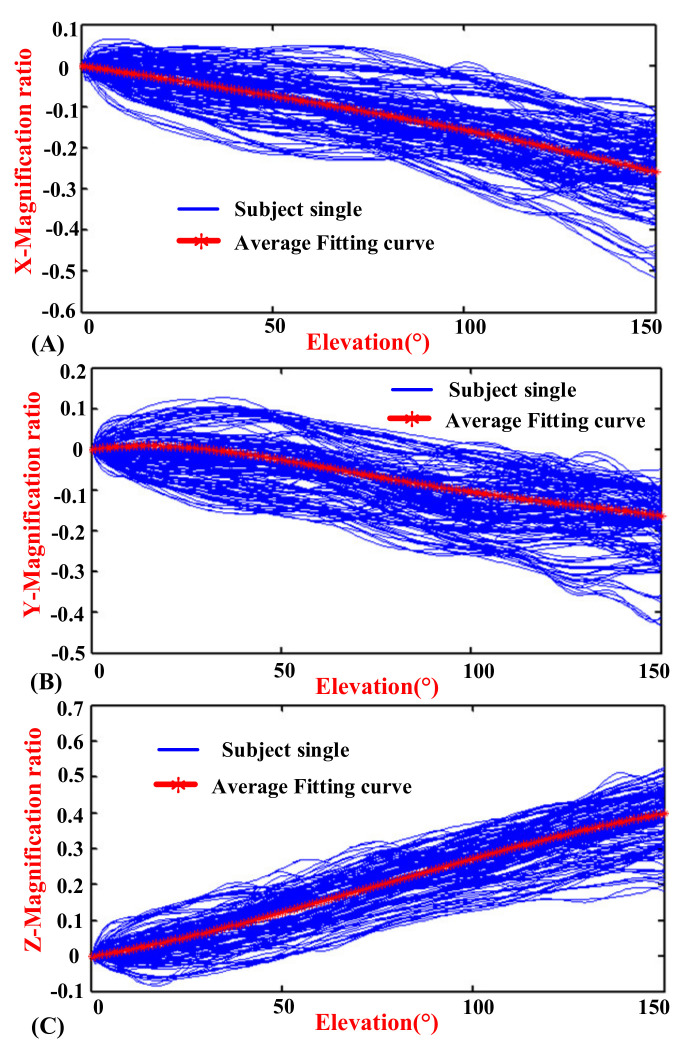
Diagrams of the magnification ratio of the GH joint center relative to the thorax coordinate system during humeral elevation in raising phase. (**A**) *X*-Magnification ratio and elevation angle. (**B**) *Y*-Magnification ratio and elevation angle. (**C**) *Z*-Magnification ratio and elevation angle.

**Figure 7:**
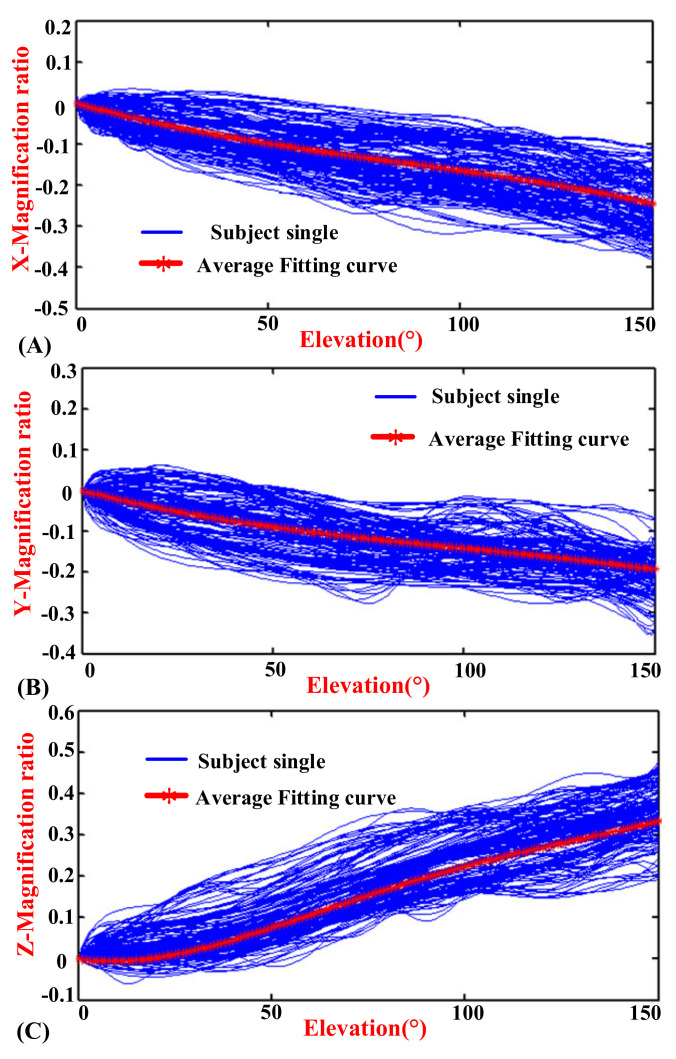
Diagrams of the magnification ratio of the GH joint center displacement variable quantity relative to the thorax coordinate system during humeral elevation in lowering phase. (**A**) *X*-Magnification ratio and elevation angle. (**B**) *Y*-Magnification ratio and elevation angle. (**C**) *Z*-Magnification ratio and elevation angle.

**Figure 8:**
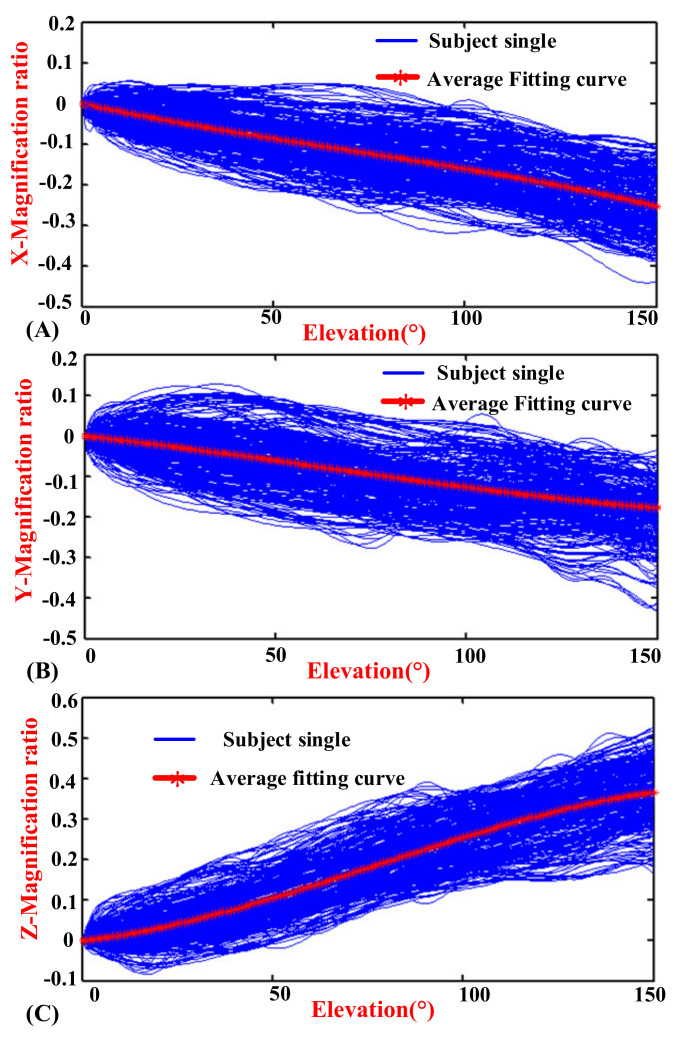
Diagrams of the magnification ratio of the GH joint center displacement variable quantity relative to the thorax coordinate system during humeral elevation in raising and lowering (RAL) phase. (**A**) *X*-Magnification ratio and elevation angle. (**B**) *Y*-Magnification ratio and elevation angle. (**C**) *Z*-Magnification ratio and elevation angle.

Figure 6, Figure 7 and Figure 8 show the motion curves (blue lines) for the twelve subjects performing humerus raising, lowering, and RAL movements in all sixteen elevation planes, respectively, with elevation angle from 0° to 150°. The movements of GH joint center displacement variable quantity changing in the *X*, *Y*, and *Z* directions during humeral elevation in different phases are observed, which are presented as (A–C) of Figure 6, Figure 7, and Figure 8, respectively. Similar patterns appear in all sixteen elevation planes relative to the humeral elevation. To summarize the motion coupling relationship, the average data points for twelve subjects performing humerus raising, lowering, and RAL movements in all sixteen elevation planes are obtained. According to the above guidelines of curve fitting in “Postprocessing of experimental data”, the polynomials of *x*, *y*, and *z* are acquired in different phases, respectively, which describe the motion coupling relationship between the *X*, *Y*, and *Z*-Magnification ratio of the GH joint center displacement variable quantity relative to the thorax coordinate system and humeral elevation angle.

In the raising phase: The degree of the polynomial *y* is four. Although the index of the RMSE is 0.005133 which is slightly more than 0.005, the motion trend of the fitting curve is very consistent with the average data. When the degree of the polynomial *y* is three, the fitting curve cannot reflect the motion rule of the average data. Although the R-square is 0.9900, the Adjusted R-square is 0.9898 and the RMSE is 0.0058, which are slightly different from the evaluation indexes of the criteria. When the degree of the polynomial *y* is five, the R-square is 0.9971, the Adjusted R-square is 0.9970, the SSE is 0.0014, and the RMSE is 0.0031, which are consistent with the evaluation indexes of the criteria. However, the fitting effect is not as good when the degree of the polynomial *y* is five as when the degree of the polynomial is four. Thus, the quartic polynomial is selected. The polynomials of *x* and *z* have shown a better fit when the degree of polynomials is three. The evaluation indexes of *X*, *Y*, and *Z*-raising in appropriate degree of the polynomials are presented in Table 1.

In the lowering phase, observing the mean data, the differences between the raising and lowering of the humerus are detected, and the fitting equations are obtained. In the polynomial of *y*, the degree of the polynomial is three. The index of the RMSE is 0.005581, which is slightly more than 0.005. The R-square is 0.9888, and Adjusted R-square is 0.9886, which are slightly less than 0.995. However, the effect of the fitting curve is closed with the average data. When the degree of the polynomial is two and four, the effect of the fitting curve is not good. Thus, the degree of the polynomial *y* is selected to be three. In the polynomial of *z*, the degree of the polynomial is four. The index of the RMSE is 0.005057, which is slightly more than 0.005. When the degree of the polynomial is three and five, the effect of the fitting curve is not good. Thus, the quartic polynomial is selected. The polynomial of *x* is effective when the degree of the polynomial is three. Lastly, the evaluation indexes of *X*, *Y,* and *Z*-lowering in appropriate degree of polynomials are also presented in Table 1.

In the RAL phase, the movement trends are different during humeral elevation in the raising and lowering phases, but the difference is not profound. Considering the application in different fields, the motion characteristics of the GH joint center displacement variable quantity during humeral elevation are also summarized. In the polynomial of *y*, the degree of the polynomial is three. The index of the RMSE is 0.005099, which is slightly more than 0.005. The R-square is 0.9914 and the Adjusted R-square is 0.9912, which are slightly less than 0.995. However, the effect of the fitting curve is good. When the degree of the polynomial is four, the SSE and RMSE are 0.001081 and 0.002722, which are less than 0.005; the R-square and Adjusted R-square are 0.995 and 0.995, respectively. However, the effect of the fitting curve is not good. Thus, the cubic polynomial is selected. The polynomials of *x* and *z* correspond well to the average data when the degree of the polynomials is three. The evaluation indexes of *X*, *Y*, and *Z*-RAL in appropriate degree of polynomials are also presented in Table 1.

Through the above analysis, the motion coupling relationship in different phases is obtained.

In the raising phase, the analytical formulas are presented by red curves in Figure 6 and named *x^r^*, *y^r^*, as well as *z^r^* in (12).

(12) $$\left\{\begin{array}{l} x^{r}=-1.279*10^{-8}*\theta^{3}-1.404*10^{-7}*\theta^{2}-1.416*10^{-3}\theta -0.001883\\ y^{r}=-1.095*10^{-9}*\theta^{4}+4.275*10^{-7}*\theta^{3}-5.57*10^{-5}*\theta^{2}+1.352*10^{-3}*\theta -0.009944\\ z^{r}=-7.088*10^{-8}*\theta^{3}+1.623*10^{-5}*\theta^{2}+1.819*10^{-3}*\theta +0.007573 \end{array}\right.$$

In the lowering phase, the analytical formulas are presented by red curves in Figure 7 and named *x^l^*, *y^l^*, as well as *z^l^* in (13).

(13) $$\left\{\begin{array}{l} x^{l}=-6.219*10^{-8}*\theta^{3}+1.577*10^{-5}*\theta^{2}-0.002594*\theta -0.006606\\ y^{l}=-4.927*10^{-8}*\theta^{3}+1.482*10^{-5}*\theta^{2}-0.002399*\theta +0.002663\\ z^{l}=1.81*10^{-9}*\theta^{4}-6.903*10^{-7}*\theta^{3}+8.636*10^{-5}*\theta^{2}-1.304*10^{-3}*\theta +0.002328 \end{array}\right.$$

In the RAL phase, the analytical formulas are presented by red curves in Figure 8 and named *x^r^*, *y^r^*, as well as *z^r^* in (14).

(14) $$\left\{\begin{array}{l} x^{RAL}=-3.749*10^{-8}*\theta^{3}+7.817*10^{-6}*\theta^{2}-2.005*10^{-3}*\theta -0.004244\\ y^{RAL}=2.487*10^{-8}*\theta^{3}-4.634*10^{-6}*\theta^{2}-1.046*10^{-3}*\theta +0.001629\\ z^{RAL}=-1.091*10^{-7}*\theta^{3}+2.518*10^{-5}*\theta^{2}+1.122*10^{-3}*\theta +0.005731 \end{array}\right.$$

### 3.2. Kinematic Model of the Shoulder Complex

The shoulder girdle is equivalent to a 3D moving platform, and the GH joint center displacement variable quantity relative to the thorax coordinate system is equal to the moving platform. Thus, the shoulder girdle can be represented as the vector ***^KK^J*** + ***q****^1^*. The humerus is represented as the vector *^K^**V***_upa_, as shown in Figure 3.

For a description of the human anatomy and clinical applications, the basic movement of the human arm is abduction/adduction (*α*) around the sagittal axis (*y*), flexion/extension (*β*) around the coronal axis (*x*), and internal/external (*γ*) around the vertical axis (*z*), which is based on the anatomical description method [45]. The elevation plane (*η*), the elevation angle (*θ*), and the internal/external rotation angle (*ψ*) are the basic variables of the ISB. For different clinical applications, they both require a transformation equation as (15).

(15) $$R_{\eta }R_{\theta }R_{\psi }=R_{\alpha }R_{\beta }R_{\gamma }$$

By using (15), the parameters of *α*, *β* and *γ* can be solved.

For specific expression, the internal/external rotation does not change the GH joint center [35,40]. During humeral elevation in different elevation planes, the movements of the shoulder girdle are accompanied by the amount of *x*, *y* and *z* in the thorax coordinate system. Thus, the humeral pointing can be expressed as (16).

(16) $$r_{E}=R_{x}.R_{y}.R_{z}.q^{1}+R_{h}.R_{\theta }.R_{\psi }.^{1}V_{upa}$$

where the point E is the midpoint of the EL and EM on the elbow. *R_η_*, *R_θ_*, and *R_ψ_* represent the rotation transformation matrix based on the ISB motion description method. *R_x_*, *R_y_*, and *R_z_* represent the mobile transformation matrix, which are the position changes of the shoulder girdle in *x*, *y*, and z directions, respectively.

Thus, the humeral pointing can be clearly described by the motion coupling relationship between the GH joint center displacement variable quantity relative to the thorax coordinate system and humeral elevation angle, and the whole kinematics of the shoulder complex has been established.

## 4. Discussion

### 4.1. Comparison of Collected Data with the Klopčar Kinematic Model

In order to verify the rationality of the experimental design and algorithm, the comparison and analysis of the collected data with previous kinematic model of the shoulder complex (Klopčar kinematic model) are performed. Firstly, the Klopčar kinematic model of the shoulder complex is described, which is a previous kinematic model to describe the coupled motion between the movements of the shoulder girdle and the humeral elevation in four elevation planes (0°, 45°, 90°, and 135°) [7,22]. Secondly, to compare with the Klopčar kinematic model, the experiment under similar conditions with Klopčar kinematic model was designed to describe the raising movement of right limb in the above-defined planes. Thus, the kinematic model of the shoulder complex, which is known as the comparative analysis kinematic (CAK)-model to distinguish this model from the above modified kinematic model, is established based on the collected data. Finally, comparison of the CAK-model and the Klopčar kinematic model is presented.

#### 4.1.1. Klopčar Kinematic Model

**Figure 9:**
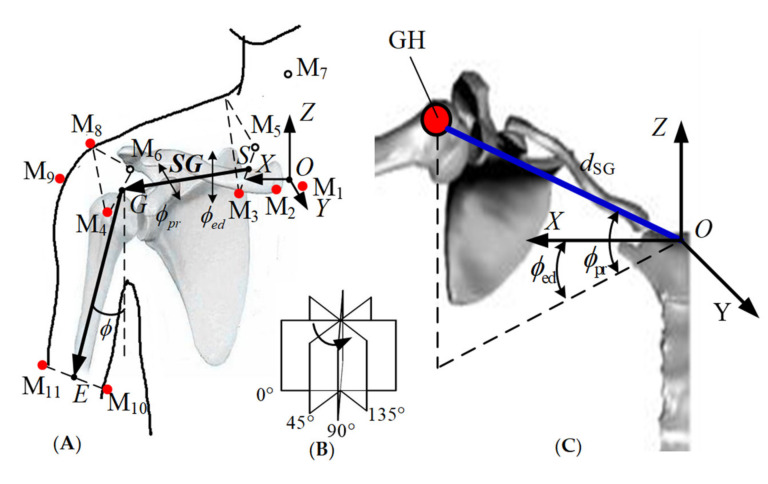
Klopčar Kinematic Model. (**A**) Reference points on the shoulder complex and the center of the GH joint in global coordinate frame. (**B**) four anatomical planes. (**C**) calculated position of the GH joint center relative to the global coordinate system.

Klopčar used the method of markers (M1–M11) pasted onto the skin of the right shoulder complex, as shown in Figure 9. The angular displacement data corresponding to the joints of the shoulder complex when the upper arm is elevated in the 0°, 45°, 90°, and 135° planes of elevation were obtained. The measurements were combined to cover the entire reachable domains of the humerus in the above-defined planes [7].

Through the analysis and integration of the data from the experiment, the coupling relationship between the movements of the shoulder girdle and humeral elevation angle was obtained. Thereinto, the angle (*ϕ_ed_*) of elevation/depression and the angle (*ϕ_pr_*) of protraction/retraction were calculated according to (17) and (18) during humeral elevation.

(17) $$\phi_{ed}=\left\{\begin{array}{ll} -0.3\phi & \phi <0^{{}^{\circ}}\\ 0 & 0^{{}^{\circ}}\leq \phi \leq 30^{{}^{\circ}}\\ 0.36\phi -10.8^{{}^{\circ}} & \phi >30^{{}^{\circ}} \end{array}\right.$$

(18) $$\phi_{pr}=\left\{\begin{array}{ll} 0.35\phi & \phi <0^{{}^{\circ}}\\ 0 & 0^{{}^{\circ}}\leq \phi \leq 70^{{}^{\circ}}\\ -0.22\phi +15.4^{{}^{\circ}} & \phi >70^{{}^{\circ}} \end{array}\right.$$

The length of the shoulder girdle (*d_SG_*) can be expressed as (19).

(19) $$d_{SG}=(-1.6\times 10^{-5}\phi +3\times 10^{-4}\phi +1)\cdot d_{0}$$

where *ϕ* is the elevation angle and *d_0_* is the shoulder girdle from S to G when the subject’s arm is hanging naturally.

#### 4.1.2. The CAK-Model

According to the description of the Klopčar kinematic model, the angular displacement data was obtained, which corresponded to the joints of the shoulder complex when the right upper arm was elevated in the above-defined planes. In this paper, the kinematic data of the shoulder complex are also obtained when the right upper arm is elevated in the above-defined planes. Other processing modes are the same as the methods and findings.

**Figure 10:**
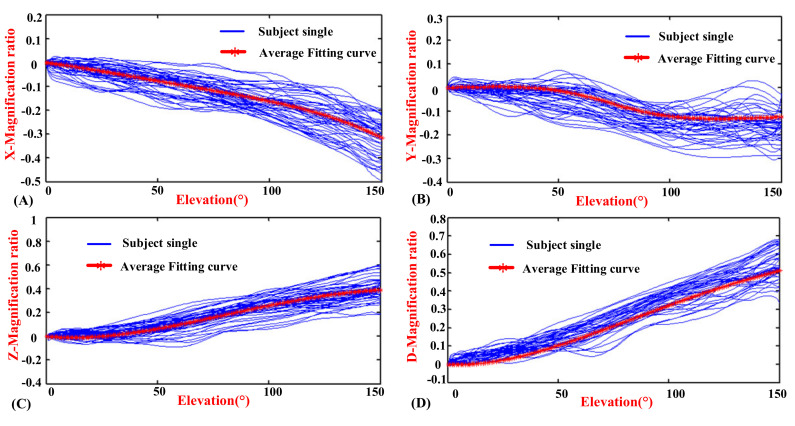
Diagrams of the magnification ratio of the GH joint center relative to the thorax coordinate system during humeral elevation. (**A**) *X*-Magnification ratio and elevation angle. (**B**) *Y*-Magnification ratio and elevation angle. (**C**) *Z*-Magnification ratio and elevation angle. (**D**) D-Magnification ratio and elevation angle.

The experimental result is shown in Figure 10, which shows the motion curves (blue lines) for the twelve subjects performing humerus raising movement in the above-defined planes, and the elevation angle is from 0° to 150°. The movements of the GH joint displacement variable quantity changing in the *X*, *Y*, and *Z* directions during humeral elevation are represented in (A), (B), and (C), respectively. To reduce unnecessary errors caused by fitting the data, the changes in length with humeral elevation are synthesized, which are represented in (D). Since similar patterns appear in the above-defined planes regarding the humeral elevation angle and to summarize the motion relationship, the average data for the twelve subjects performing humerus raising movements in the above-defined planes are obtained.

According to the above criteria for establishing the fitting curve, the *Y*-Magnification ratio of the GH joint center displacement variable quantity and humeral elevation angle does not meet the above conditions of the polynomial fit. Observing the average data, the rational fitting method was applicative, and the numerator degree and denominator degree of the fitting equation are both two. Although the evaluation indexes are slightly different from that of the criteria, the fitting effect is good. Thus, the fitting equation is selected. In addition, the other average data is consistent with the motion rule of the polynomials. Just as that method yields analytical Equations (12)–(14), the polynomials of *x*, *z*, and *d* are acquired, which describe the *X*, *Z*, and *D*-Magnification ratio of the GH joint center displacement variable quantity and humeral elevation angle in the raising phase, respectively. In the polynomial of *z*, the degree of the polynomials is four. The indexes of SSE and RMSE are 0.005915 and 0.006365, respectively, which are slightly more than 0.005. However, by analyzing the trend of the fitting curves, the equation curve agrees well with the average data. When the degree of the polynomial is five, the SSE and RMSE are 0.0008807 and 0.002465, which are less than 0.005. However, the motion trend of the fitting curve is similar to that when the degree of the polynomial is four, which can be regarded as overfitting. When the degree of the polynomial is three, the fitting curve cannot reflect the motion rule of the average data. Thus, the quartic polynomial is selected. For the same result, the polynomial of *d* is obtained. The polynomial of *x* has shown a better fit when the degree of polynomial is four. The result of the fitting equation curves agrees with the average data; the elevation indexes of *x*, *y*, *z*, and *d*-CAK are also presented in Table 1.

According to the above analysis, the motion coupling relationship is presented, and the analytical formulas presented as the red curves in Figure 10 are named *x^CAK^*, *y^CAK^*, *z^CAK^*, and *d^CAK^* as (20).

(20) $$\left\{\begin{array}{l} x^{CAK}=-8.585*10^{-10}*\theta^{4}+1.783*10^{-7}*\theta^{3}-1.343*10^{-5}*\theta^{2}-1.201*10^{-3}*\theta -0.002929\\ y^{CAK}=(-7.101*10^{-2}*\theta^{2}+3.798*\theta -83.28)./(\theta^{2}-147.4*\theta +7895)-0.001055\\ z^{CAK}=5.108*10^{-10}*\theta^{4}-4.124*10^{-7}*\theta^{3}+7.918*10^{-5}*\theta^{2}-1.714*10^{-3}*\theta +0.001467\\ d^{CAK}=7.435*10^{-10}*\theta^{4}-4.161*10^{-7}*\theta^{3}+7.25*10^{-5}*\theta^{2}-6.134*10^{-4}*\theta +0.002263 \end{array}\right.$$

#### 4.1.3. Comparison of the CAK-Model and the Klopčar Kinematic Model

For the Klopčar kinematic model, the origin of the global coordinate system was the crossing of an axis (medial and lateral) through the GH joint center and an axis (anterior and posterior) through the thorax. The calculated reference point was the center of the M3 and M5 markers. However, the locations are not clear, leading to difficult estimates. However, the distance from the center of the sternum to point G is three-quarters of the initial distance from that, which was presented in [22], and the result is applied. Considering the different coordinate frames, we unify the the GH joint displacement variable quantity relative to the thorax coordinate system. Thus, the Klopčar kinematic model is transformed into (21) from (17) to (19).

Humerus posture vector:

(21) $$\left\{\begin{array}{l} \begin{array}{l} x(\phi )=0.75*(d_{SG}\cdot \cos\phi_{ed}\cdot \cos\phi_{pr}-1)\\ y(\phi )=0.75*d_{SG}\cdot \cos\phi_{ed}\cdot \sin\phi_{pr}\\ z(\phi )=0.75*d_{SG}\cdot \sin\phi_{ed} \end{array}\\ d(\phi )=\sqrt{x{\left(\phi \right)}^{2}+y{\left(\phi \right)}^{2}+z{\left(\phi \right)}^{2}} \end{array}\right.$$

**Figure 11:**
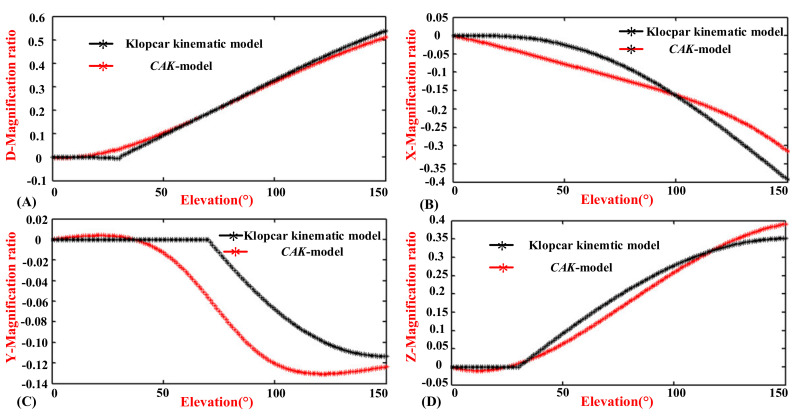
Comparison of the CAK-model and the Klopčar Kinematic Model. (**A**) Translational displacement variable quantity *d*^CAK^ and *d*(Φ). (**B**) Translational displacement variable quantity *x*^CAK^ and *x*(Φ). (**C**) Translational displacement variable quantity *y*^CAK^ and *y*(Φ). (**D**) Translational displacement variable quantity *z*^CAK^ and *z*(Φ).

According to (20) and (21), the *x*(*ϕ*), *y*(*ϕ*), *z*(*ϕ*), and *d*(*ϕ*) are zero when the elevation angle is zero. For the unity of the results, the constant terms of the *d^CAK^*, *x^CAK^*, *y^CAK^,* and *z^CAK^* of the GH joint center displacement variable quantity are ignored, which are shown in (A), (B), (C), and (D) of Figure 11. For further comparison, the displacement variable quantity *d*(*ϕ*), *x*(*ϕ*), *y*(*ϕ*), and *z*(*ϕ*) of the Klopčar kinematic model is added.

In Figure 11A, the trajectory of displacement variable quantity *d^CAK^* is smoothly aligned with the angle *ϕ* from 0° to 150°, although the gradient of the trajectory is different. The curve has a sharp point when the angle of elevation is 30°. The reason for this is that Klopčar assumed that the lengths of elongation and shortening are basically the same, while the *X* direction changes slightly. The rest of the curve is essentially consistent with that of *d*(*ϕ*). However, two curves have a slightly different gradient, especially when the angle is close to 150°.

In Figure 11B, the displacement variable quantity *x^CAK^* is reduced uniformly with the angle *ϕ* from 0° to 120°. However, the decrease in the gradient with the increase of the angle is from 120° to 150°. Furthermore, the GH joint center displacement variable quantity *x*(*ϕ*) along the axis *x* moves slightly with the angle *ϕ* from 0° to 40°. The gradient and angle present a negative correlation from 40° to 150°. The curve of *x^CAK^* is not entirely consistent with that of *x*(*ϕ*), but the trend is basically the same.

In Figure 11C, the displacement variable quantity *y^CAK^* changes slightly with the angle *ϕ* from 0° to 50°, and the gradient of the curve decreases from 50° to 100°. Then, the gradient of the curve rises slightly with the angle *ϕ* from 100° to 150°. Furthermore, the GH center displacement variable quantity *y*(*ϕ*) along the *y* axis stays in the same position with the angle *ϕ* from 0° to 70°. The gradient of the curve decreases from 70° to 120°, the change in which is very evident. From 120° to 150°, the gradient of the curve does not change significantly. The reason that the two curves are visually obvious is because the spacing of the *y*-axis is set to be only 0.02, and we can see that the maximum gap ratio between the Klopčar kinematic model and CAK-model is only 0.05 and the trend is consistent.

In Figure 11D, the displacement variable quantity *z^CAK^* changes steadily with the angle *ϕ* from 0° to 30°. However, the change is very small. The increases in displacement variable quantity *z^CAK^* are first large and then small with the angle *ϕ* from 30° to 150°. In addition, the displacement variable quantity *z*(*ϕ*) along the *z* axis does not move from the angle *ϕ* from 0° to 30°, which matches *z^CAK^*. From 30° to 150°, the displacement *z*(*ϕ*) and *z^CAK^* are almost the same.

### 4.2. Comparison of the Motion Coupling Characteristics in Different Phases

The shoulder girdle has a large range of self-redundant characteristics. When the humerus has a fixed elevation, the different positions of the GH joint center are presented [7,40,46]. Klopčar analyzed the redundant characteristics of the shoulder girdle named as the shoulder girdle angular motion. In the raising and lowering phases of the humerus, the coupled motion differences of the shoulder girdle were within the range of the angular motion [7]. In this study, the different movement trends of the GH joint center displacement variable quantity are found during humeral elevation in the raising, lowering, and RAL phases, under the same experimental conditions, which is thought to be self-motion; in other words, the GH joint moves with the functional relevance of the shoulder girdle during humeral elevation [2].

Since the self-motion of the shoulder girdle is more concerned about the daily motion process (not concerned about the initial motion) of human upper limb in this paper, this discussion only exposits the self-motion of the shoulder girdle during humerus elevation in different phases (the premise is that the GH joint center displacement variable quantity in different phases is considered to be zero when the elevation angle is zero), and the self-redundant characteristics have been confirmed when the arm is nonelevated in [7]. Based on the above conditions, the self-motion of the shoulder girdle during humerus elevation in different phases is analyzed.

**Figure 12:**
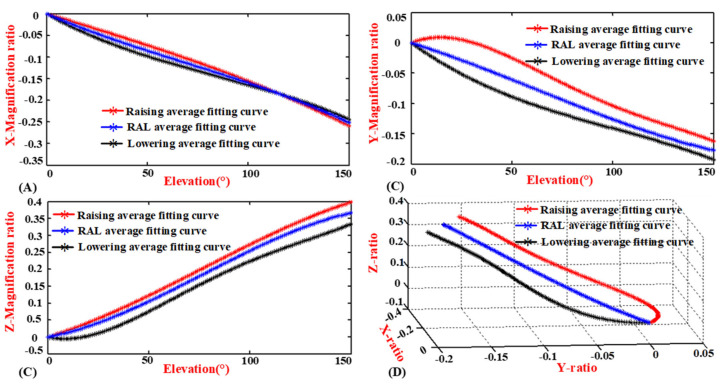
Comparison of the Motion Coupling Characteristics in Different Phases. (**A**) *X*-direction magnification ratio and humeral elevation angle. (**B**) *Y*-direction magnification ratio and humeral elevation angle. (**C**) *Z*-direction magnification ratio and humeral elevation angle. (**D**) *X*, *Y*, and *Z*-directions magnification ratio.

The changes in the *X*-direction are shown in Figure 12A. During humeral elevation in the raising phase, the gradient is relatively small in the initial stage, and then the gradient increases. When humeral elevation is in the lowering phase, the gradient is large in the initial stage, and then the gradient decreases. They intersect in the middle, and then they basically follow the same gradient. In the end, they basically overlap. The RAL curve is located between the black and red curves. However, the three curves differ slightly among the humeral elevations.

The changes in the *Y*-direction are shown in Figure 12B. The gradient of the humeral elevation in the raising phase is essentially unchanged in the initial stage. Subsequently, this gradient gradually increases. The gradient of the humeral elevation in the lowering stage is large in the initial stage. Subsequently, this gradient gradually and slowly decreases. In the last stage, it gradually maintains the same gradient. However, the motion range of the shoulder girdle in the raising phase is less than that in the lowering phase of the humeral elevation, and the difference in the magnification ratio is 0.04 when the elevation angle is 150°. Similarly, the RAL curve is located between the black and red curves. However, the three curves differ greatly among the humeral elevation in different phases.

The changes in the *Z*-direction are shown in Figure 12C. In the raising phase, the magnification ratio and elevation angle are positively correlated during humeral elevation from 0° to 150°, except for a weak correlation in the initial and last stages. In the lowering phase, the magnification ratio is essentially unchanged in the initial stage. The magnification ratio gradually increases in the intermediary stage. The magnification ratio and elevation angle are negatively correlated in the last stage. However, the motion range of the shoulder girdle in the lowering phase is less than that in the raising phase of the humeral elevation, for which the final magnification ratio difference is 0.076. Similarly, the RAL curve is located between the red and black curves.

Figure 12D shows the *X-*, *Y-,* and *Z*-directions magnification ratio of the GH joint center displacement variable quantity with humeral elevation from 0° to 150° in different phases. According to the 3D perspective, the movement trends are different, and the motion curve with humeral elevation in the RAL phase is always enveloped by the red convex curve and the black concave curve. Additionally, the three curves are different in both their appearance pattern and magnitude.

Since the influence of detection accuracy and measurement error under the same experimental conditions are similar, their motion trend is not the same through the above analysis, which confirms the “self-motion” of the shoulder girdle.

### 4.3. Discussion of the Whole Paper

The shoulder complex has a certain coupling relationship between humerus and the components of the shoulder girdle, which is a crucial problem in the kinematic model of the shoulder complex. In order to establish a proper kinematic model of the shoulder complex, the first step is to establish a mechanism model of the shoulder complex. Various kinematic models of the shoulder complex have been proposed in recent years. The commonality is to simplify the GH joint into a spherical joint, and the difference is the simplification of the shoulder girdle’s kinematic model. To model the shoulder complex, it is not enough just to think about the mechanism model—the joint coupling motion relationship and the limiting of the coupling motion should also be considered.

Some researchers, who obtained the exact information of the sternum, clavicle, scapula, and humerus using the intra cortical pins, X-ray, and computed tomography, studied the coupling motion relationship of the shoulder complex. Unfortunately, the motion coupling relationship of the shoulder complex is in a certain particular position or defined plane. Other researchers synthesized the coupled motion between the movements of the shoulder girdle and the humeral elevation by estimating the instantaneous rotation center of the GH joint through the average position of three marker points and the movements of the shoulder girdle. Since the complexity of the shoulder complex lies in the movement of shoulder girdle and the invisibility of the GH joint center [7,10,22], it is also difficult to quantify and understand the whole coupled motion of the shoulder complex [7,22].

In this work, considering the whole coupled motion of the shoulder complex, a mechanism model has been presented, which is a generalized GH joint with floating center. For the GH joint center displacement variable quantity relative to the thorax coordinate system is equal to the overall motion of the shoulder girdle; the coupled motion can clearly describe the characteristics of the shoulder girdle and humerus. For specific expression, the internal/external rotation does not change the GH joint center [35,40]. Thus, the motion coupling relationship between the GH joint center displacement variable quantity and the humeral elevation can clearly describe the overall motion characteristics of the shoulder complex. To go a step further, an algorithm was designed, the experiment was setup, and the experimental data were processed. Finally, the motion coupling relationship was quantified. Considering the effect of skin deformation, the accuracy of measuring scapula and humerus is affected to some extent. The GH joint center displacement variable quantity relative to the thorax coordinate system is large (a few centimeters) and using motion capture technology to acquire detection data based on skin markers can meet the precision requirements of human daily movement science.

Observing the Figure 6, Figure 7 and Figure 8, the GH joint center displacement variable quantity appeared to be slightly larger for the effect of the deviation of the markers pasted on acromion from bone target during arm elevation [43], the difference of the arm lifted movement in the sixteen elevation plane, and also the self-motion of the shoulder girdle [7,40,46]. The mean curve was fitted to obtain the modified model of the shoulder complex. To go a step further, the comparison and analysis of the collected data with the previous kinematic model also verified the rationality of the experimental design, algorithm, and postprocessing of experimental data. For a large range of self-redundant characteristics of the shoulder girdle, the differences of the motion coupling relationship in different phases are also analyzed. The significance of raising and lowering being separate can quantify the differences of the motion coupling relationship and provide a reference for further accurate study of self-redundant characteristics of the shoulder girdle. Considering the application in different fields, the motion characteristics of the GH joint center displacement variable quantity during humeral elevation in RAL phase are also summarized, which has practical significance for the daily movement of human upper limb. In addition, since the shoulder joint motion coupling relationship between genders has no essential difference [7,22], it was not studied here.

The experimental results indicate the motion coupling relationship between the GH joint center displacement variable quantity and the humeral elevation in the raising, lowering, and RAL phases relative to the sternum, which are also consistent with the results of other researchers [22,40,46]. However, it should be highlighted that, due to the effect of the deviation of the skin markers from bone target during movement of the arm and the simplification of GH joint as a spherical joint, the kinematic model is only applied to provide basic data for the design of upper limb rehabilitation robots, shoulder function simulation, and ergonomics. Although the slippage of the markers on the scapula was only 4.2 mm during full elevation of the arm [38] and the GH joint center displacement relative to the scapula was 12.4 mm during full elevation of the arm [47], it cannot be used in joint replacement, prosthesis design, and other areas which need accurate kinematic information. The exact model of the shoulder complex using the pin inserted into the sternum, clavicle, scapula, and humerus will be addressed in our future work.

Nevertheless, according to the results obtained in this study, the model provides basic data for the design of upper limb rehabilitation robots and has practical significance for shoulder function simulation, the daily movement of human upper limb, and ergonomics.

## 5. Conclusions and Future Work

In this study, the motion coupling relationships between the GH joint center displacement variable quantity relative to the thorax coordinate system and the humeral elevation angle in the raising phase, lowering phase, and RAL phase were investigated using markers captured by the Vicon motion system. Analytical formulas were acquired and a modified kinematic model of the shoulder complex was quantified, which can be regarded as a generalized GH joint with floating center. The comparison of the collected data to the Klopčar kinematic model shows a similar trend in the description of the motion coupling relationship, which provides strong evidence that the modified kinematic model is effective. Additionally, the differences in the motion coupling relationships between the GH joint center displacement variable quantity and the humeral elevation angle in different phases confirm the self-motion of the shoulder girdle. Compared with commercial software, such as LifeMOD, AnyBody, and OpenSim, the advantages of the reported work are that using healthy subjects with different ages, heights, weights, and body types to conduct the experiment by using Vicon motion capturing system is more in line with the actual situation of the human body structure, and the description of overall motion characteristics of shoulder complex was validated by comparing the results with a prior kinematic model from literature, which shows that the obtained modified kinematic model of the shoulder complex is accurate enough for the design of upper limb rehabilitation robots.

Future work will focus on increasing the sample database of human shoulder movements to reduce the impact of individual differences on the measurement data and using the pin inserted into the sternum, clavicle, scapula, as well as humerus to study the exact coupling motion relationship.

## Figures and Tables

**Table 1:** Indexes of fitting curve for modified and CAK-model.

	SSE	R-Square	Adjusted R-Square	RMSE
*X*-raising	0.001932	0.9977	0.9976	0.003625
*Y*-raising	0.003847	0.9922	0.992	0.005133
*Z*-raising	0.0008856	0.9996	0.9996	0.002455
*X*-lowering	0.0008811	0.9986	0.9986	0.002448
*Y*-lowering	0.004578	0.9888	0.9886	0.005581
*Z*-lowering	0.003733	0.998	0.998	0.005057
*X*-RAL	0.0003742	0.9995	0.9995	0.001595
*Y*-RAL	0.003822	0.9914	0.9912	0.005099
*Z*-RAL	0.002489	0.9988	0.9988	0.004114
*x*-CAK	0.0001855	0.9998	0.9998	0.001127
*y*-CAK	0.006171	0.9871	0.9868	0.006502
*z*-CAK	0.005195	0.998	0.998	0.006502
*d*-CAK	0.008561	0.998	0.9979	0.007557
